# High-Level γ-Glutamyl-Hydrolase (GGH) Expression is Linked to Poor Prognosis in ERG Negative Prostate Cancer

**DOI:** 10.3390/ijms18020286

**Published:** 2017-01-29

**Authors:** Nathaniel Melling, Masoud Rashed, Cornelia Schroeder, Claudia Hube-Magg, Martina Kluth, Dagmar Lang, Ronald Simon, Christina Möller-Koop, Stefan Steurer, Guido Sauter, Frank Jacobsen, Franziska Büscheck, Corinna Wittmer, Till Clauditz, Till Krech, Maria Christina Tsourlakis, Sarah Minner, Hartwig Huland, Markus Graefen, Lars Budäus, Imke Thederan, Georg Salomon, Thorsten Schlomm, Waldemar Wilczak

**Affiliations:** 1 Institute of Pathology, University Medical Center Hamburg-Eppendorf, Hamburg D-20246, Germany; n.melling@uke.de (N.M.); masoudrashed@msn.com (M.R.); cor.schroeder@uke.de (C.S.); c.hube@uke.de (C.H.-M.); m.kluth@uke.de (M.K.); d.lang@uke.de (D.L.); c.koop@uke.de (C.M.-K.); s.steurer@uke.de (S.S.); g.sauter@uke.de (G.S.); f.jacobsen@uke.de (F.J.); f.buescheck@uke.de (F.B.); c.wittmer@uke.de (C.W.); t.clauditz@uke.de (T.C.); t.krech@uke.de (T.K.); m.tsourlakis@uke.de (M.C.T.); s.minner@uke.de (S.M.); w.wilczak@uke.de (W.W.); 2 General, Visceral and Thoracic Surgery Department and Clinic, University Medical Center Hamburg-Eppendorf, Hamburg D-20246, Germany; 3 Martini-Clinic, Prostate Cancer Center, University Medical Center Hamburg-Eppendorf, Hamburg D-20246, Germany; hhuland@uke.de (H.H.); graefen@uke.de (M.G.); budaeus@uke.de (L.B.); i.thederan@uke.de (I.T.); gsalomon@uke.de (G.S.); t.schlomm@uke.de (T.S.); 4 Department of Urology, Section for translational Prostate Cancer Research, University Medical Center Hamburg-Eppendorf, Hamburg D-20246, Germany

**Keywords:** GGH, ERG, deletion, prostate cancer, TMA, folic acid, tissue biomarkers, prognostic factors, multivariate models

## Abstract

γ-glutamyl-hydrolase (GGH) is a ubiquitously-expressed enzyme that regulates intracellular folate metabolism for cell proliferation, DNA synthesis, and repair. Employing GGH immunohistochemistry on a tissue microarray with 12,427 prostate cancers, we found that GGH expression was negative to low in normal prostate epithelium, whereas 88.3% of our 10,562 interpretable cancers showed GGH expression. GGH staining was considered as low intensity in 49.6% and as high intensity in 38.6% of cancers. High GGH expression was linked to the TMPRSS2:ERG-fusion positive subset of cancers (*p* < 0.0001), advanced pathological tumor stage, and high Gleason grade (*p* < 0.0001 each). Further analysis revealed that these associations were merely driven by the subset of ERG-negative cancers, High GGH expression was weakly linked to early biochemical recurrence in ERG negative cancers (*p* < 0.0001) and independent from established histo-pathological parameters. Moreover, GGH expression was linked to features of genetic instability, including presence of recurrent deletions at 3p, 5q, 6q, and 10q (PTEN, *p* ≤ 0.01 each), as well as to accelerated cell proliferation as measured by Ki67 immunohistochemistry (*p* < 0.0001). In conclusion, the results of our study identify GGH as an ERG subtype specific molecular marker with modest prognostic relevance, which may have clinical relevance if analyzed in combination with other molecular markers.

## 1. Introduction

Prostate cancer is the most prevalent cancer in men in Western societies [1]. Although most cancers have an indolent clinical course, this disease represents the third most common cause of cancer death in men. A reliable distinction between indolent and aggressive forms of the disease is highly desirable for therapeutic decision-making. Despite recent advances, the established pre-treatment prognostic parameters currently include Gleason grade and tumor extent on biopsies, preoperative prostate-specific antigen (PSA), and clinical stage. Since these parameters are statistically powerful, but not always sufficient for optimal individual treatment decisions, it can be hoped that a further understanding of disease biology will eventually lead to clinically better applicable molecular tests that enable a reliable prediction of prostate cancer aggressiveness.

γ-Glutamyl-hydrolase (GGH) is a highly-conserved, ubiquitously-expressed lysosomal glycoprotein involved in folate metabolism [2,3,4]. GGH affects the intracellular concentration of polyglutamylated folic acid by catalyzing the hydrolysis of polyglutamylated folate into transportable monoglutamate. Polyglutamylated folic acid is an essential cofactor of various folate-dependent enzymes with a role in DNA metabolism [5,6]. For example, adequate folate levels are essential for synthesis of the nucleic acid thymidine and of the common methyl donor S-adenosyl methionine, which is required for the maintenance of DNA methylation patterns [7]. Up-regulation of GGH has been described to occur in several neoplasias, including breast and colon cancers, pulmonary neuroendocrine tumors, and hematological cancers [8,9,10,11], and has been linked to adverse tumor features and poor clinical outcome in some of them [8,11]. GGH overexpression is also of interest for anti-cancer therapies, as it was found to confer resistance to fluorouracil (5-FU)-based chemotherapies in several cancers [4,12,13,14]. Studies on GGH expression and its potential impact on prostate cancer biology are currently lacking.

Given the increasing evidence for a role of GGH in cancer, we became interested in its role in prostate cancer. We, therefore, studied the expression of GGH by immunohistochemistry on a large tissue microarray (TMA) including more than 12,400 prostate cancers. Comparison with extensive pathological and clinical follow-up data, as well as molecular data on key molecular alterations of prostate cancer enabled us to draw conclusion on its clinical relevance and on possible molecular mechanisms it might affect in this disease.

## 2. Results

### 2.1. Technical Issues

A total of 10,562 (85%) tumor samples were interpretable in our TMA analysis. Reasons for non-informative cases (1865 spots; 15%) included lack of tissue samples or absence of unequivocal cancer tissue in the TMA spot.

### 2.2. GGH Expression in Normal Prostate Tissue and Prostate Cancer

**Figure 1:**
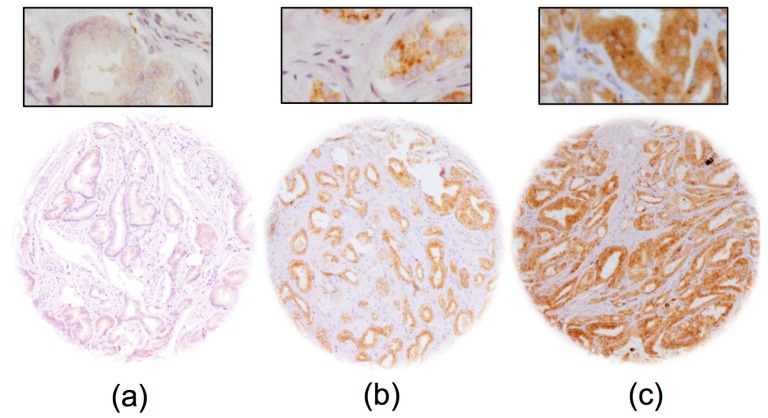
Representative images of GGH staining in prostate cancer with (**a**) negative; (**b**) low and (**c**) high intensity staining. Magnification 100×; insert 400×; TMA spot size 600 μm.

Normal prostate glands showed absent to low cytoplasmic staining in luminal cells under the selected experimental conditions. GGH staining was detectable in 9322 of our 10,562 (88.3%) interpretable prostate cancers, and was considered as “low intensity” in 49.6% and as “high intensity” in 38.6% of cancers. Representative images of GGH staining are given in Figure 1.

### 2.3. Association with TMPRSS2:ERG Fusion Status and ERG Protein Expression

**Figure 2:**
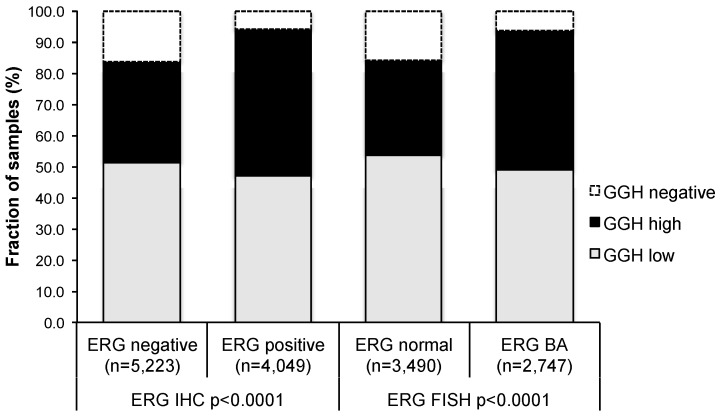
Association between GGH staining and ERG status as determined by IHC and FISH analysis.

To evaluate whether GGH expression is associated with ERG status in prostate cancers, we used data from previous studies (expanded from [15,16]). Data on TMPRSS2:ERG fusion status obtained by fluorescence in situ hybridization (FISH) was available from 6237 and by immunohistochemistry from 9272 tumors with evaluable GGH staining. Data on both ERG FISH and immunohistochemistry (IHC) were available from 5978 cancers, and an identical result (ERG IHC-positive and break by FISH, or ERG IHC-negative and missing break by FISH) was found in 5712 of 5978 (95.6%) cancers. High-level GGH staining was linked to TMPRSS2:ERG rearrangement and ERG positivity in prostate cancers. For example, high GGH staining was seen in 47.2% and 44.6% of cancers with TMPRSS2: ERG fusion detected by IHC and FISH, but was found in only 32.4% of cancers without ERG staining, and in 30.6% of cancers without ERG rearrangements (*p* < 0.0001 each; Figure 2).

### 2.4. Association with Tumour Clinical Characteristics

GGH expression levels were only marginally related to prostate cancer clinical characteristics if all cancers were jointly analyzed. For example, high-level GGH staining was found in 37% of pT2 cancers and in 41% of pT3b-pT4 cancers, or in 31% of Gleason ≤ 3 + 3 and 39% of Gleason ≥ 4 + 4 tumors (*p* < 0.0001 each, Table 1). Since GGH showed differential expression in ERG-positive and ERG-negative cancers, we also analyzed both subsets separately. This analysis revealed that significant associations between GGH and adverse cancer clinical characteristics mainly existed in the subset of ERG negative cancers. Here, high-level GGH expression was strongly linked to advanced pathological tumor stage (*p* = 0.0016) and high Gleason grade (*p* < 0.0001; Table S1), albeit the differences in absolute numbers between subgroups were not large. These associations between GGH expression and tumor clinical characteristics were weaker in the subset of ERG-positive cancers (Table S2). That significant *p* values were obtained in some analyses is obviously due to the high number of analyzed samples.

**Figure 3:**
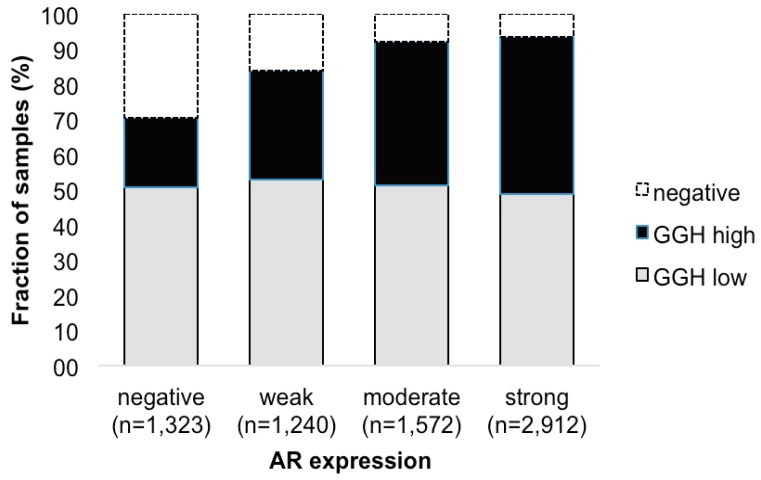
Association between positive GGH staining and AR expression.

Androgen receptor (AR) expression was strongly linked to GGH staining (Figure 3). High-level GGH expression was found in 19.9% of cancers without detectable AR expression, but in 44.6% of tumors with strong AR expression (*p* < 0.0001, each).

### 2.5. Association with Androgen Receptor

Androgen receptor (AR) expression was strongly linked to GGH staining (Figure 3). High-level GGH expression was found in 19.9% of cancers without detectable AR expression, but in 44.6% of tumors with strong AR expression (*p* < 0.0001, each).

### 2.6. Association with Other Key Genomic Deletions

**Figure 4:**
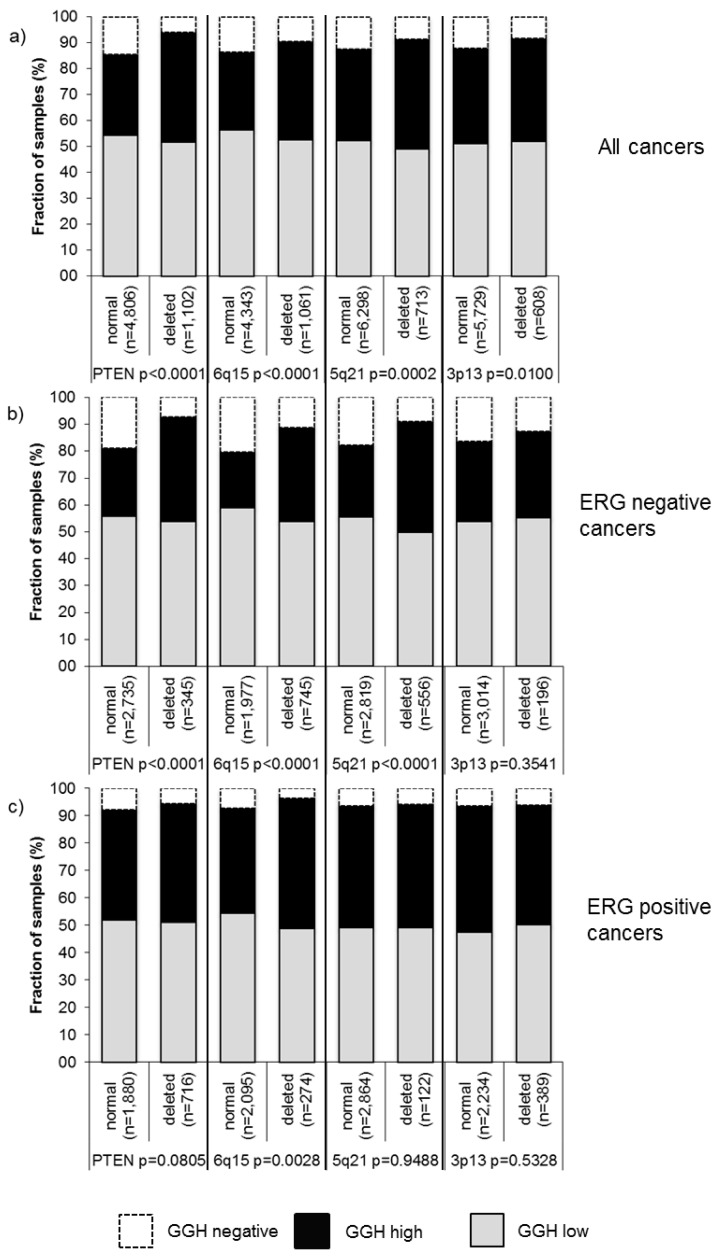
Association between positive GGH staining and 10q23 (PTEN), 5q21 (CHD1), 6q15 (MAP3K7), 3p13 (FOXP1) deletions in (**a**) all cancers; (**b**) ERG-negative; and (**c**) ERG-positive subset.

Earlier studies have provided evidence for distinct molecular subgroups of prostate cancers defined by TMPRSS2:ERG fusions and several genomic deletions. Others, as well as ourselves, have previously described a strong link of PTEN and 3p13 deletions to ERG positivity and of 5q21 and 6q15 deletions to ERG negativity [17,18,19,20]. To study whether GGH expression might be particularly associated with one of these genomic deletions, GGH data were compared to pre-existing findings on 10q23 (PTEN), 3p13 (FOXP1), 6q15 (MAP3K7), and 5q21 (CHD1) deletion. If all cancers were jointly analyzed, GGH expression was significantly linked to all deletions (PTEN (*p* < 0.0001), 3p13 (*p* = 0.0100), 5q21 (*p* = 0.0002), 6q15 (*p* < 0.0001); Figure 4a). Subgroup analysis of ERG-negative and ERG-positive cancers revealed that these associations were merely driven by the subset of ERG-negative cancers (Figure 4b), while there was no unequivocal impact of GGH levels on the deletion status in ERG-positive cancers (Figure 4c).

### 2.7. Association with Tumor Cell Proliferation

Strong GGH staining was significantly linked to high cell proliferation, as measured by the Ki67 labeling index (LI). The average Ki67LI increased from 2.0 ± 0.1 in cancers lacking GGH expression to 2.7 ± 0.05 in cancers with low and to 3.1 ± 0.06 in cancers with high GGH levels (*p* < 0.0001). This association held true in most tumor subsets with identical Gleason score (≤3 + 3: *p* = 0.005, 3 + 4: *p* < 0.0001, 4 + 3: *p* = 0.001, ≥4 + 4: *p* = 0.2015).

### 2.8. DNA Ploidy Status

**Figure 5:**
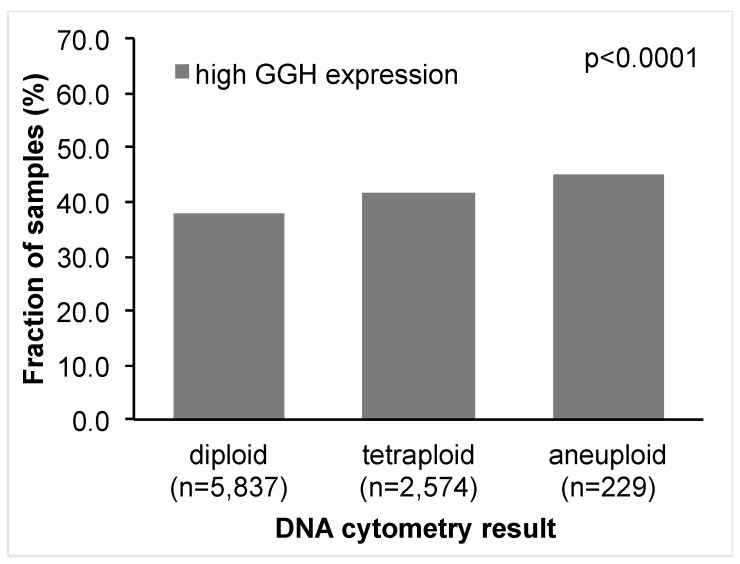
Association between DNA ploidy status and GGH expression.

GGH expression was significantly associated with DNA ploidy status (*p* < 0.0001, Figure 5). High GGH expression was seen in 2212 of 5837 (38%) patients with diploid DNA status, in 42% of patients with tetraploid DNA status, and in 45% of patients with aneuploid DNA status.

### 2.9. Association with PSA Recurrence

**Figure 6:**
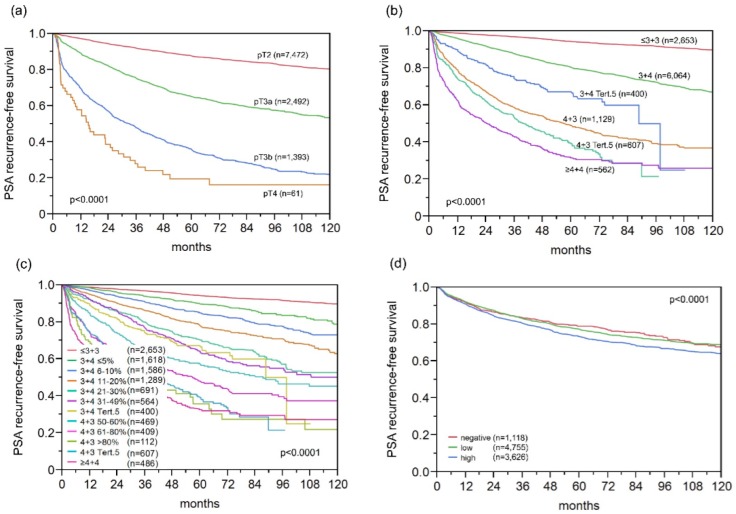

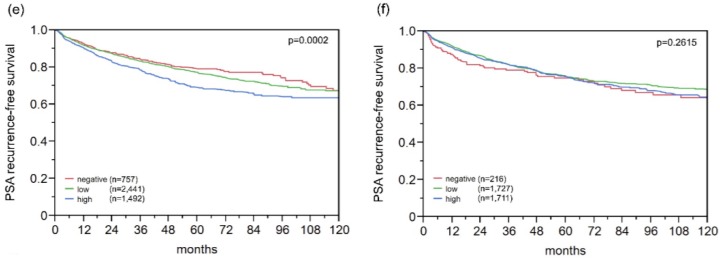
Biochemical recurrence after radical prostatectomy and (**a**) pathological tumor stage; (**b**) classical Gleason score; (**c**) quantitative Gleason score; GGH expression in (**d**) all cancers; (**e**) ERG fusion-negative; and (**f**) ERG fusion-positive cancers.

Follow-up data were available for 9875 patients with interpretable GGH staining on the TMA. The prognostic impact of pT stage (Figure 6a), traditional Gleason grade (Figure 6b), and quantitative Gleason grade (Figure 6c) were strongly linked to PSA recurrence. A weak, but significant, association between high-level GGH expression and early PSA recurrence was found if all cancers were jointly analyzed (*p* < 0.0001, Figure 6d). Subset analyses revealed a similar 10-year PSA recurrence-free survival of 62% for high GGH expression in ERG negative and positive cancers (Figure 6e,f). Analyzing subsets of tumors with comparable traditional and quantitative Gleason grades revealed that GGH expression levels largely lacked prognostic impact in morphologically-defined tumor subsets (Figure S1). A prognostic impact of GGH expression was only seen in Gleason 4 + 3 = 7 cancers (*p* = 0.0265, Figure S1a), but this association disappeared if Gleason 4 + 3 = 7 cancers were further subdivided according to their percentage of Gleason 4 fractions (Figure S1b–h).

### 2.10. Multivariate Analysis

Four different scenarios of multivariate analyses were performed to evaluate the clinical relevance of GGH expression (Table 2). Scenario 1 evaluated the postoperatively available parameters (pathological tumor stage, pathological lymph node status (pN), surgical margin status, preoperative PSA value, and pathological Gleason grade obtained after the morphological evaluation of the entire resected prostate). In Scenario 2, the postoperatively available parameters with exception of nodal status were included. The rationale for this approach was that excluding pN in multivariate analysis could markedly increase case numbers. This is because indication and extent of lymph node dissection is not standardized in radical prostatectomy. Two additional scenarios should reflect the preoperative situation. Scenario 3 included GGH expression, preoperative PSA, clinical tumor stage (cT stage), and Gleason grade obtained on the prostatectomy specimen. Since postoperative determination of a tumors Gleason grade is “better” than the preoperatively-determined Gleason grade (subjected to sampling errors and, consequently, under-grading in more than one third of cases [21]), another multivariate analysis was added. Scenario 4 included the preoperative Gleason grade obtained on the original biopsy combined with preoperative PSA, cT stage, and GGH expression. GGH turned out to be an independent prognostic parameter in all four scenarios when all tumors were jointly analyzed (*p* < 0.03 each, Table 2). This held also true for ERG-negative, but not for ERG-positive, cancers (Table 2).

## 3. Discussion

The results of this study show that a high level of γ-glutamyl-hydrolase is associated with early PSA recurrence in ERG-negative prostate cancers. Our immunhistochemical analysis showed detectable GGH staining in 88.3% of 10,562 interpretable prostate cancers. Others have so far not examined GGH in prostate cancer but the high fraction of GGH-positive tissue samples is well in line with the ubiquitous expression of GGH described in human tissues [22,23,24]. That about 40% of our cancers showed stronger GGH staining than normal prostate glands suggests that GGH may become upregulated during prostate cancer development and/or progression. Tumor-associated upregulation of GGH has indeed been reported also for various other human cancers, including, for example, urothelial carcinoma of the bladder, breast, lung, or colorectal cancer [8,11,25,26]. Given the central need for GGH in DNA replication, it can be assumed that increased cell proliferation—a typical feature of malignant tumors—should go along with higher levels of GGH in the respective cells. The significant association found in this study between GGH expression and rapid tumor cell proliferation supports this concept.

GGH overexpression was weakly linked to adverse features of prostate cancer in our study, including advanced tumor stage, high Gleason grade, and early biochemical recurrence. That GGH expression increased only slightly with tumor stage and Gleason grade supports a role of GGH upregulation already in early stages of prostate cancer. Interestingly, the largest increase in GGH expression occurred at the transition from Gleason 3 + 3 to Gleason 3 + 4. It is, thus, tempting to speculate that GGH upregulation may occur in early prostate cancer progression.

To generate hypotheses on the molecular mechanisms associated with GGH upregulation during prostate cancer development and progression we exploited the molecular database attached to our TMA. The relationship between GGH expression, androgen receptor (AR) expression, and ERG activation was analyzed because of the central role of AR for prostate cancer, and because ERG activation is the most frequent molecular alteration in this tumors. About 50% of prostate cancers carry a gene fusion involving the androgen-regulated serine protease TMPRSS2 and the ETS-transcription factor ERG. This fusion results in a strong AR-dependent activation of ERG with subsequent transcriptional deregulation of more than 1600 ERG target genes. We found that both ERG activation and AR levels were paralleled by a marked increase of GGH expression, raising the hypothesis that GGH upregulation may be triggered by AR signaling (Figure 4). Of note, hormone-dependent regulation of GGH is also supported by a recent study showing strong associations of GGH and estrogen/progesterone receptor levels in breast cancer [8].

In addition, GGH upregulation was linked to features of genetic instability, such as the presence of chromosomal deletions. This observation fits well to the important role of folate for maintenance of genome integrity [7]. It has been shown that excess levels of GGH remove folic acid from the cell by converting it from its polyglutamylated form—which cannot pass the cell membrane—to a transportable monoglutamylated derivate [4,9]. Under conditions of folate deficiency, uracil is synthesized and incorporated in the DNA instead of thymidine [27], a process that has been associated with the generation of point mutations, single- and double-strand DNA breaks, and chromosome breakage [28,29]. In addition, folate deficiency has been linked to impaired mitochondrial metabolism with accumulation of DNA-damaging reactive oxygen species [30,31,32], and recent work further suggests that GGH-dependent reduction of the intracellular folic acid concentration may also induce epigenetic alterations [9,33], which can impact genetic integrity [34,35,36,37].

In previous studies using the same TMA, we identified various proteins for which expression was at higher levels in ERG-positive than in ERG-negative prostate cancers. In several of these, a prognostic impact was only seen for ERG-negative, but not for ERG-positive cancers [38,39,40]. The present study demonstrates that GGH belongs to this type of proteins. Other biomarkers were only prognostic in ERG-positive cancers [41,42,43]. Overall, these data suggest that tumor-relevant functions of GGH and other proteins can become attenuated or amplified in an ERG-positive molecular environment. This is conceivable as ERG activation leads to a substantial modification of the intracellular environment with significant changes in the expression of more than 1600 genes [44,45,46]. The frequent ERG dependence of prognostic biomarkers in prostate cancer challenges the development and use of prognostic molecular test that are applicable to all patients [47,48]. It appears probable that different tests need to be developed for molecular subtypes such as for example ERG positive and ERG negative cancers. Given that the prognostic difference between cancers with negative/low or high GGH expression was only about 10%, GGH does not seem to be strong prognostic marker if analyzed on its own. Given its statistical independence of established prognostic features, it cannot be excluded, that GGH expression measurement may aid in decision-making in ERG-negative prostate cancers if combined with other markers.

It is of note that the Gleason grade is the strongest (and least expensive) prognostic feature in prostate cancer. In a recent analysis we have demonstrated that by using the percentage of unfavorable Gleason patterns, the Gleason grading can be transformed from a categorical into a continuous variable with an even finer distinction of prognostic subgroups [49]. That the prognostic impact of GGH expression largely disappeared in groups defined by Gleason grade categories or by comparable percentages of Gleason 4 patterns demonstrates the power of morphologic malignancy assessment. These findings show that the requirements for a molecular test to be clearly better than morphology are rather high.

Higher GGH activity has been associated with 5-FU resistance in several tumor entities [50,51,52,53]. 5-FU plays only a minor part in the chemotherapeutical approach in prostate cancer. In advanced castration-resistant prostate cancers, 5-FU has been used alone or in combination with other chemotherapeutic drugs and/or radiation as palliative therapy, but the response rates in prostate cancer typically do not exceed about 20% [54]. Finding GGH expression in about 90% of our prostate cancers may provide an explanation for the poor response against 5-FU-based therapies.

## 4. Materials and Methods

### 4.1. Patients

Radical prostatectomy specimens were available from 12,427 patients, undergoing surgery between 1992 and 2012 at the Department of Urology and the Martini Clinics at the University Medical Centre Hamburg-Eppendorf. Archived diagnostic leftover tissues was used for manufacturing of tissue microarrays and analyzed for research purposes approved by the local ethics committee (Ethics commission Hamburg, WF-049/09 and PV3652). According to local laws (HmbKHG, §12,1), informed consent was not required for this study. Patient records/information was anonymized and de-identified prior to analysis. All work has been carried out in compliance with the Helsinki Declaration. Histopathological data were retrieved from the patients’ records, including tumor stage, Gleason grade, nodal stage and stage of the resection margin. In addition to the classical Gleason categories, “quantitative” Gleason grading was performed as described before [49]. In brief, for every prostatectomy specimen, the percentages of Gleason 3, 4, and 5 patterns were recorded in cancerous tissues as part of the regular process of Gleason grading. Gleason 3 + 4 and 4 + 3 cancers were subdivided according to their percentage of Gleason 4. For practical use, we subdivided the 3 + 4 and 4 + 3 cancers in eight subgroups: 3 + 4 ≤ 5% Gleason 4, 3 + 4 6%–10%, 3 + 4 11%–20%, 3 + 4 21%–30%, 3 + 4 31%–49%, 4 + 3 50%–60%, 4 + 3 61%–80% and 4 + 3 > 80% Gleason 4. In addition, separate groups were defined by the presence of a tertiary Gleason 5 pattern, including 3 + 4 Tertiary 5 and 4 + 3 Tertiary 5. Follow-up data were available for 12,344 patients with a median follow-up of 50 months (range: 1–264 months; Table S3). Prostate specific antigen (PSA) values were measured following surgery and PSA recurrence was defined as a postoperative PSA of 0.2 ng/mL and increasing at first of appearance. All prostate specimens were analyzed according to a standard procedure, including a complete embedding of the entire prostate for histological analysis [55]. The TMA manufacturing process was described earlier in detail [56,57]. In short, one 0.6 mm core was taken from a representative tissue block from each patient. The tissues were distributed among 27 TMA blocks, each containing 144–522 tumor samples. For internal controls, each TMA block also contained various control tissues, including normal prostate tissue. The molecular database attached to this TMA contained results on androgen receptor expression (*n* = 7047) [15], ERG expression in 10,678 [15], ERG break-apart fluorescence in situ hybridization (FISH) analysis in 7099 (expanded from [16]), and deletion status of 5q21 (CHD1) in 7932 (expanded from [18]), 6q15 (MAP3K7) in 6069 (expanded from [18]), 10q23 (PTEN) in 6704 (expanded from [17]), and 13p13 (FOXP) in 7081 (expanded from [18]) cancers and DNA ploidy status in 10,087 cancers (expanded from [58]). 

### 4.2. Immunohistochemistry

Freshly-cut TMA sections were immunostained on one day and in one experiment. Slides were deparaffinised and exposed to heat-induced antigen retrieval for 5 min in an autoclave at 121 °C in pH 7.8 Tris-EDTA-citrate buffer. A primary antibody specific for GGH (rabbit polyclonal antibody, Sigma-Aldrich, Munich, Germany; cat#HPA025226; dilution 1:450) was applied at 37 °C for 60 min. The bound antibody was then visualized using the EnVision Kit (Dako, Glostrup, Denmark) according to the manufacturer’s directions. GGH typically stained the cytoplasm in all (100%) tumor cells of a tissue spot. Accordingly, the staining intensity was recorded in three categories for each cancer, including negative (no detectable staining), low (weak to moderate staining), and high (strong staining) by independent evaluation by at least two scorers.

### 4.3. Statistics

For statistical analysis, MP 12.0 software (SAS Institute Inc., Cary, NC, USA) was used. Contingency tables were calculated to study the association between GGH expression and clinico-pathological variables, and the chi-squared (likelihood) test was used to find significant relationships. Kaplan–Meier curves were generated using biochemical (PSA) recurrence as the clinical endpoint. The log-rank test was applied to test the significance of differences between stratified survival functions. Cox proportional hazards regression analysis was performed to test the statistical independence and significance between pathological, molecular, and clinical variables.

## 5. Conclusions

In summary, the results of our study demonstrate that GGH is another example for an ERG subtype-specific molecular marker. The small prognostic difference between cancers with high or low GGH expression limits its clinical impact as a stand-alone marker. However, GGH may be a suitable cofactor if combined with other molecular markers.

## Figures and Tables

**Table 1:** Association between GGH staining results and prostate cancer clinical characteristics in all cancers.

Parameter	GGH IHC Result (%)				*p* Value
	N Evaluable	Negative	Low	High	
**All cancers**	10,562	11.7	49.6	38.6	
**Tumor stage**					
pT2	6851	12.3	50.8	36.9	<0.0001
pT3a	2333	11.0	46.5	42.5		
pT3b-pT4	1338	9.9	49.1	41.0		
**Gleason grade**					
≤3 + 3	2329	16.7	52.3	31.0	<0.0001
3 + 4	5430	10.8	49.1	40.1		
3 + 4 Tertiary 5	397	10.1	51.9	38.0		
4 + 3	914	10.2	48.1	41.7		
4 + 3 Tertiary 5	568	8.1	48.2	43.7		
≥4 + 4	450	12.0	48.9	39.1		
**Lymph node metastasis**					
N0	5996	10.3	48.0	41.7	0.0928
N+	608	12.2	50.3	37.5		
**Preoperative PSA level (ng/mL)**					
<4	1290	9.5	50.5	39.9	<0.0001
4–10	6362	11.3	48.9	39.9		
10–20	2076	12.6	51.4	36.0		
>20	726	17.1	49.9	33.1		
**Surgical margin**					
Negative	8413	11.7	49.9	38.4	0.9259
Positive	1949	11.9	49.4	38.7		

**Table 2:** Multivariate analysis including GGH expression in prostate cancers, the ERG-negative, and ERG-positive subset in different clinical scenarios.

Subset	Scenario	*N* Analyzable	*p* Value							
			Preoperative PSA-Level	pT Stage	cT Stage	Gleason Grade RPE ^1^	Gleason Grade Biopsy	N-Stage	R-Status	GGH-Expression
Total	1	5773	<0.0001	<0.0001	-	<0.0001	-	<0.0001	0.0010	0.0290
	2	9281	<0.0001	<0.0001	-	<0.0001	-	-	<0.0001	0.0047
	3	9163	<0.0001	-	<0.0001	<0.0001	-	-	-	0.0246
	4	9038	<0.0001	-	<0.0001	-	<0.0001	-	-	0.0032
ERG neg. subset	1	2928	0.0002	<0.0001	-	<0.0001	-	0.0006	0.0894	0.0094
	2	4583	<0.0001	<0.0001	-	<0.0001	-	-	0.0006	0.0111
	3	4549	<0.0001	-	<0.0001	<0.0001	-	-	-	0.1042
	4	4492	<0.0001	-	<0.0001	-	<0.0001	-	-	0.0197
ERG pos. subset	1	2255	0.0060	<0.0001	-	<0.0001	-	0.0038	0.0143	0.4035
	2	3573	<0.0001	<0.0001	-	<0.0001	-	-	<0.0001	0.2573
	3	3500	<0.0001	-	<0.0001	<0.0001	-	-	-	0.1868
	4	3447	<0.0001	-	<0.0001	-	<0.0001	-	-	0.2427

^1^ Radical prostatectomy.

## Supplementary Materials

Supplementary materials can be found at www.mdpi.com/1422-0067/18/2/286/s1.

## Abbreviations

AR	Androgen receptor
CHD1	Chromodomain-Helicase-DNA-Binding Protein 1
FISH	Fluorescence in-situ hybridization
FOXP1	Forkhead box protein P1
GGH	γ-Glutamyl-Hydrolase
IHC	Immunohistochemistry
Ki67LI	Ki67 labeling index
MAP3K	Mitogen-Activated Protein Kinase Kinase Kinase 7
PSA	Prostate specific antigen
PTEN	Phosphatase and tensin homolog
RPE	Radical prostatectomy
TMPRSS2:ERG	Transmembrane protease, serine 2: ETS-related gene fusion
TMA	Tissue microarray
