# Supplementary material for: High-Level γ-Glutamyl-Hydrolase (GGH) Expression is Linked to Poor Prognosis in ERG Negative Prostate Cancer
Source: Int J Mol Sci. 2017 Jan 29;18(2):286. doi: 10.3390/ijms18020286 (PMC5343822; doi:10.3390/ijms18020286)

Figure A1 Prognostic impact of GGH expression in subsets of cancers defined by the Gleason score. a) Impact of low (red line) and high (blue line) GGH expression as compared to the classical Gleason score categories (indicated by black dotted lines). b-h) Impact of low (red line) and high (blue line) GGH expression as compared to the quantitative Gleason score categories (black dotted lines) defined by subsets of cancers with b) ≤5% Gleason 4 patterns, c) 6-10% Gleason4 patterns, d) 11-20% Gleason 4 patterns, e) 21-30% Gleason 4 patterns, f) 31-49 % Gleason 4 patterns, g) 50-60% Gleason 4 patterns, h) 61-100% Gleason 4 patterns.

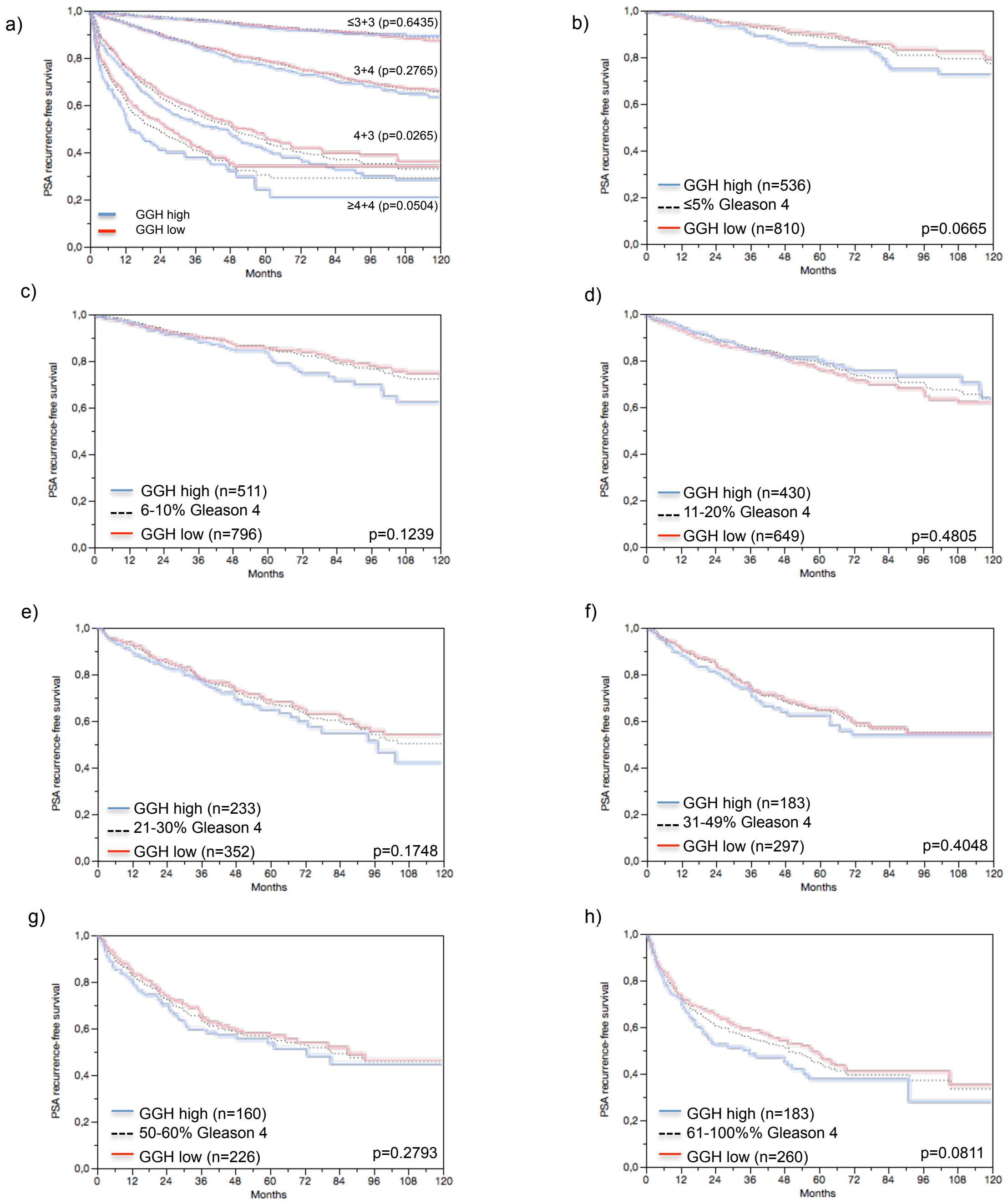

Supplement: Supplementary file 1 [file ijms-18-00286-s001.zip › FigureA1.pdf]
