# Supplementary material for: High-Level γ-Glutamyl-Hydrolase (GGH) Expression is Linked to Poor Prognosis in ERG Negative Prostate Cancer
Source: Int J Mol Sci. 2017 Jan 29;18(2):286. doi: 10.3390/ijms18020286 (PMC5343822; doi:10.3390/ijms18020286)
Supplement: Supplementary file 1 [file ijms-18-00286-s001.zip › TableA1.pdf]

Table A1. Association between GGH immunostaining results and prostate cancer phenotype in ERG fusion **negative** tumours.

		GGH IHC result (%)			
**Parameter**	**n evaluable**	**Negative**	**Low**	**High**	**p value**
**All cancers**	5,223	16.1	51.5	32.4	
**Tumour stage**					0.0016
pT2	3,506	16.9	52.1	30.9	
pT3a	1,049	15.6	50.3	34.0	
pT3b-pT4	652	12.1	50.5	37.4	
**Gleason grade**					<0.0001
≤3+3	1,065	23.9	53.8	22.3	
3+4	2,681	14.9	51.0	34.2	
3+4 Tert.5	239	13.0	56.5	30.5	
4+3	482	13.3	51.5	35.3	
4+3 Tert.5	307	10.4	50.8	38.8	
≥4+4	263	15.2	46.0	38.8	
**Lymph node metastasis**					0.2423
N0	3,049	14.2	51.1	34.7	
N+	290	13.4	46.9	39.7	
**Preoperative PSA level (ng/ml)**					0.0166
<4	551	13.8	52.3	33.9	
4-10	3,104	15.8	50.8	33.5	
10-20	1,114	15.9	54.6	29.5	
>20	411	20.9	48.4	30.7	
**Surgical margin**					0.7265
Negative	4,175	16.2	51.9	31.9	
Positive	953	15.7	51.0	33.3	
