# Supplementary material for: High-Level γ-Glutamyl-Hydrolase (GGH) Expression is Linked to Poor Prognosis in ERG Negative Prostate Cancer
Source: Int J Mol Sci. 2017 Jan 29;18(2):286. doi: 10.3390/ijms18020286 (PMC5343822; doi:10.3390/ijms18020286)
Supplement: Supplementary file 1 [file ijms-18-00286-s001.zip › TableA2.pdf]

Table A2. Association between GGH immunostaining results and prostate cancer phenotype in ERG fusion **positive** tumours.

		GGH (%)			
**Parameter**	**n evaluable**	**Negative**	**Low**	**High**	**p value**
**All cancers**	4,049	5.9	46.9	47.2	
**Tumour stage**					0.0104
pT2	2,411	5.4	48.5	46.1	
pT3a	1,072	6.2	43.2	50.7	
pT3b-pT4	551	8.0	46.8	45.2	
**Gleason grade**					0.1031
≤3+3	842	7.8	47.5	44.7	
3+4	2,176	5.3	47.4	47.2	
3+4 Tert.5	126	4.8	39.7	55.6	
4+3	350	5.7	44.3	50.0	
4+3 Tert.5	218	4.6	46.3	49.1	
≥4+4	135	8.9	49.6	41.5	
**Lymph node metastasis**					<0.0001
N0	2,317	5.4	44.3	50.3	
N+	259	11.6	53.7	34.7	
**Preoperative PSA level (ng/ml)**					0.0015
<4	551	4.2	48.6	47.2	
4-10	2,473	5.5	45.9	48.6	
10-20	731	7.3	47.3	45.4	
>20	249	10.4	51.8	37.8	
**Surgical margin**					0.5931
Negative	3,174	5.8	46.8	47.4	
Positive	800	6.4	48.1	45.5	
