# Supplementary material for: High-Level γ-Glutamyl-Hydrolase (GGH) Expression is Linked to Poor Prognosis in ERG Negative Prostate Cancer
Source: Int J Mol Sci. 2017 Jan 29;18(2):286. doi: 10.3390/ijms18020286 (PMC5343822; doi:10.3390/ijms18020286)
Supplement: Supplementary file 1 [file ijms-18-00286-s001.zip › TableA3.pdf]

Table A3. Pathological and clinical data of the arrayed prostate cancers. Percentage in the column “Study cohort on TMA” refers to the fraction of samples across each category. Percentage in column “Biochemical relapse among categories” refers to the fraction of samples with biochemical relapse within each parameter in the different categories.

	No. of patients (%)	
	Study cohort on TMA (n=12427)	Biochemical relapse among categories
**Follow-up (mo)**		
n	11665 (93.9%)	2769 (23.7%)
Mean	62.9	-
Median	50.0	-
Age (y)		
≤50	334 (2.7%)	81 (24.3%)
51-59	3061 (24.8%)	705 (23%)
60-69	7188 (58.2%)	1610 (22.4%)
≥70	1761 (14.3%)	370 (21%)
**Pretreatment PSA (ng/ml)**		
<4	1585 (12.9%)	242 (15.3%)
4-10	7480 (60.9%)	1355 (18.1%)
10-20	2412 (19.6%)	737 (30.6%)
>20	812 (6.6%)	397 (48.9%)
**pT stage (AJCC 2002)**		
pT2	8187 (66.2%)	1095 (13.4%)
pT3a	2660 (21.5%)	817 (30.7%)
pT3b	1465 (11.8%)	796 (54.3%)
pT4	63 (0.5%)	51 (81%)
**Gleason grade**		
≤3+3	2848 (22.9%)	234 (8.2%)
3+4	6679 (53.8%)	1240 (18.6%)
3+4 Tert.5	433 (3.5%)	115 (26.6%)
4+3	1210 (9.7%)	576 (47.6%)
4+3 Tert.5	646 (5.2%)	317 (49.1%)
≥4+4	596 (4.8%)	348 (58.4%)
**pN stage**		
pN0	6970 (91%)	1636 (23.5%)
pN+	693 (9%)	393 (56.7%)
**Surgical margin**		
Negative	9990 (81.9%)	1848 (18.5%)
Positive	2211 (18.1%)	853 (38.6%)

NOTE: Numbers do not always add up to 12,427 in the different categories because of cases with missing data. Abbreviation: AJCC, American Joint Committee on Cancer.
